# Application of PI3K inhibitors in breast cancer treatment: a clinical trial landscape analysis based on clinical trial databases and registries

**DOI:** 10.3389/fonc.2026.1807951

**Published:** 2026-04-13

**Authors:** Junjie Cao, Yanru Chen, Jiani Song, Shuang Li, Yalin Tang, Xinru Zhang, Lichuan Zhang, Lin Ma

**Affiliations:** 1 Department of Emergency, Affiliated Hospital of North Sichuan Medical College, Nanchong, Sichuan, China; 2 School of Clinical Medicine, North Sichuan Medical College, Nanchong, Sichuan, China; 3 School of Imaging Medicine, North Sichuan Medical College, Nanchong, Sichuan, China

**Keywords:** breast cancer, clinical trial, efficacy and safety, PI3K inhibitors, targeted therapy

## Abstract

**Objective:**

To characterize the clinical trial landscape of PI3K inhibitors in breast cancer and evaluate their efficacy, safety, and publication status.

**Methods:**

We searched eight major clinical trial databases using standardized MeSH/Emtree terms up to January 1, 2026. Of 283 potentially eligible studies screened independently by two reviewers, 87 trials were included for descriptive statistical analysis using R 4.5.1 and SPSS 26.0. Inter-rater agreement was assessed using Cohen’s kappa.

**Results:**

The United States led global research activity, Europe formed a collaborative network, and China and Korea emerged as important nodes in the Asia-Pacific region. Phase I trials accounted for 34.5% of included studies. PI3Kα was the predominant target (46 trials), and alpelisib was the most extensively studied agent. Marked publication bias was observed, with over 60% of trials involving key targets remaining unpublished; phase I trials had the lowest reporting rate. PI3Kα inhibitors, particularly alpelisib and inavolisib, combined with endocrine therapy improved progression-free survival in PIK3CA-mutated HR+/HER2− advanced breast cancer, while alpelisib was the only agent to demonstrate an overall survival benefit. Pan-PI3K inhibitors showed more limited efficacy and greater toxicity. Hyperglycemia and diarrhea were the most commonly reported serious adverse events.

**Conclusion:**

PI3Kα inhibitors show promising efficacy in PIK3CA-mutated HR+/HER2− breast cancer, but publication bias, resistance, target-specific toxicity, and geographic disparities remain major barriers. Mandatory trial reporting, biomarker-guided treatment, and international collaboration should be prioritized.

## 1 Introduction

Breast cancer remains the most common malignancy among women worldwide and a major cause of cancer-related death, imposing a substantial burden on public health and healthcare systems (1). According to the systematic analysis of the 2023 Global Burden of Disease Study, an estimated 2.3 million new cases of breast cancer occur each year (2.3 million [95% UI, 2.03–2.61]), and the number of deaths exceeds 680,000 (778,000 [683,000–870,000]), with the incidence continuing to increase in both developed and developing countries (2025). Although significant progress has been made in comprehensive treatment strategies, including surgery, chemotherapy, radiotherapy, endocrine therapy, and targeted therapy, the prognosis for patients with advanced or metastatic breast cancer remains unsatisfactory. Moreover, studies have found that the median survival of patients with lung metastasis who do not receive radiotherapy is only 12–15 months, and the 5-year survival rate is less than 10% (2). Tumor heterogeneity, drug resistance, and treatment-induced adverse reactions remain major challenges in clinical practice, underscoring the urgent need to explore new therapeutic targets and effective drugs (3).

The phosphatidylinositol 3-kinase/protein kinase B/mammalian target of rapamycin (PI3K/AKT/mTOR) signaling pathway is one of the most commonly dysregulated pathways in human cancers, including breast cancer (4). This pathway plays a crucial role in regulating fundamental cellular processes such as cell proliferation, survival, migration, invasion, and metabolism, which are closely associated with tumor initiation, progression, and resistance (5). In breast cancer, abnormal activation of the PI3K pathway is mainly driven by genetic variations, including mutations in PIK3CA (the gene encoding the catalytic subunit PI3Kα), loss of function of PTEN (the negative regulator of this pathway), and overexpression of PI3K or AKT (6). It has been reported that approximately 40% of patients with breast cancer harbor PIK3CA mutations, especially in the hormone receptor-positive (HR+) subtype (7), making the PI3K pathway a promising therapeutic target for the treatment of breast cancer.

In recent years, PI3K inhibitors have emerged as a new class of targeted agents for cancer treatment, and several have been approved by the U.S. Food and Drug Administration (FDA) or have entered late-stage clinical trials (**Table 1**) (7–11). These inhibitors exert antitumor effects by specifically blocking activation of the PI3K pathway, thereby inhibiting tumor cell proliferation and inducing apoptosis (7). For example, alpelisib is a selective PI3Kα inhibitor that has received FDA approval for the treatment of advanced breast cancer harboring PIK3CA mutations in patients with HR+/human epidermal growth factor receptor 2-negative (HER2-) disease (12). However, the clinical efficacy of PI3K inhibitors varies significantly among different breast cancer subtypes, and their use is further limited by drug resistance (13) and severe adverse reactions (such as hyperglycemia, diarrhea, and rash). As the first approved selective PI3Kα inhibitor, alpelisib demonstrated significant efficacy in the phase III SOLAR-1 clinical trial. However, target-specific toxicities, mainly hyperglycemia and diarrhea, led to treatment discontinuation or dose reduction in up to 40% of patients because of adverse reactions, severely limiting its clinical application (14). Additionally, a randomized adaptive phase II/III study found that the pan-PI3K inhibitor buparlisib showed significantly increased off-target toxicity because of its lack of subtype specificity, and its efficacy did not meet expectations in multiple phase III clinical trials, ultimately failing to gain approval for breast cancer indications (15). Although the mutation rate of PIK3CA in HR+/HER2- breast cancer is as high as 40–50%, the real-world detection rate is only 20–30% (16), far below the clinical demand. The resistance mechanisms of PI3K inhibitors are complex and diverse, and differences between preclinical studies and real-world settings make it difficult to formulate clinical treatment strategies (17).

**Table 1:** Summary of information on FDA-approved PI3K inhibitor drugs.

Drug	Target	FDA approval year	Approved indications	Type/stage of breast cancer	Benefits	Adverse effects	Drug market status	The number of clinical trials on clinicaltrials.gov
Idelalisib	PI3Kδ	2014	Blood system tumors (CLL, FL, SLL)	\	1-year progression-free survival (PFS):66% (ZYDELIG + R) vs 13% (rituximab alone) Median PFS:19.4 months (95% CI: 12.3 months-NR) vs 6.5 months (95% CI: 4.0-7.3 months); HR = 0.15 (95% CI: 0.09-0.24), P<0.0001 Overall response rate (ORR):84% (all PR, 95% CI: 75%-90%) vs 16% (all PR, 95% CI: 9%-24%), P<0.0001 Median duration of response (DOR):Not reached (95% CI: 12 months-NR) vs 6.2 months (95% CI: 2.8-6.5 months)	The most common adverse reactions (≥30%) with ZYDELIG included diarrhea, pneumonia, pyrexia, fatigue, rash, cough, and nausea. In combination with rituximab, the most frequent serious adverse reactions (SARs) were pneumonia (23%), diarrhea (10%), pyrexia (9%), sepsis (8%), and febrile neutropenia (5%).	Normally available for sale	79
Copanlisib	PI3K(pan)	2017	FL	\	The objective response rate was 58.7% (95% CI: 48.6%-68.2%) with an estimated median response duration of 12.2 months (range, 0+ to 22.6 months). The complete response rate was 14.4% and partial response rate was 44.2%.	Common adverse reactions in greater than 20% of patients include hyperglycemia, diarrhea, fatigue, hypertension, leukopenia, neutropenia, nausea, lower respiratory tract infections, and thrombocytopenia. The most common grade 3–4 adverse reactions include hyperglycemia, leukopenia, hypertension, neutropenia, and lower respiratory tract infections. Serious non-infectious pneumonitis occurred in 6% of patients.	In 2023, Withdrawn cancer accelerated approvals	75
Duvelisib	PI3Kδ/γ	2018	Blood system tumors (CLL, FL, SLL)	\	In patients with CLL/SLL, treatment with COPIKTRA resulted in a 60% lower risk of progression compared with ofatumumab.Median progression-free survival (PFS) was 16.4 months in the COPIKTRA group versus 9.1 months in the ofatumumab group, representing a 7.3-month improvement in PFS.In patients who received at least two prior therapies, the overall response rate (ORR) was more than 70%, with all responses being partial responses.	Fatal adverse reactions within 30 days of the last dose occurred in 12% of patients treated with COPIKTRA versus 4% in those treated with ofatumumab. Serious adverse reactions were reported in 73% of patients receiving COPIKTRA, most frequently infection (38%) and diarrhea or colitis (23%). Common adverse reactions in greater than 20% of patients include diarrhea or colitis, neutropenia, pyrexia, upper respiratory tract infection, pneumonia, rash, fatigue, nausea, anemia, and cough.	Normally available for sale	62
Alpelisib	PI3Kα	2019	Breast cancer associated with PIK3CA gene mutation	Adult patients diagnosed with locally advanced or metastatic breast cancer characterized by HR+, HER2− status and PIK3CA mutational activation	In patients with PIK3CA-mutated breast cancer, the median progression-free survival (PFS) was 11.0 months (95% CI: 7.5–14.5 months) with PIQRAY + fulvestrant versus 5.7 months (95% CI: 3.7–7.4 months) with placebo + fulvestrant (HR = 0.65; 95% CI: 0.50–0.85; P = 0.0065). The objective response rate (ORR) was 35.7% (95% CI: 27.4%–44.7%) in patients with measurable disease and 26.6% (95% CI: 20.1%–34.0%) in all PIK3CA-mutated patients, compared with 16.2% (95% CI: 10.4%–23.5%) and 12.8% (95% CI: 8.2%–18.7%) in the placebo arm, respectively. The median overall survival (OS) was 39.3 months (95% CI: 34.1–44.9 months) versus 31.4 months (95% CI: 26.8–41.3 months) (HR = 0.86; P = 0.15), and PIQRAY + fulvestrant delayed the median time to symptomatic deterioration by 8.5 months (23.3 months vs 14.8 months; HR = 0.72; 95% CI: 0.54–0.95).	The most common adverse reactions (any grade, ≥20%) with PIQRAY + fulvestrant were hyperglycemia (79.2%), diarrhea (59.5%), rash (51.8%), lymphopenia (55.3%), elevated gamma-glutamyl transferase (53.2%), elevated creatinine (67.6%), fatigue (43.3%), anemia (44.0%), elevated alanine aminotransferase (44.0%), elevated lipase (42.6%), nausea (46.8%), decreased appetite (35.9%), stomatitis (30.3%), vomiting (28.5%), weight loss (27.8%), and alopecia (20.4%). The most frequent grade 3/4 adverse reactions included hyperglycemia (39.1%), rash (19.4%), gamma-glutamyl transferase elevation (12.0%), lymphopenia (9.2%), diarrhea (7.0%), lipase elevation (7.0%), fatigue (5.6%), and weight loss (5.3%).	Normally available for sale	127
Umbralisib	PI3Kδ/CK1ϵ	2021	FL and MZL	\	For marginal zone lymphoma (MZL), the overall response rate (ORR) was 49.3% (95% CI: 37.0%-61.6%) with a complete response (CR) rate of 15.9% and partial response (PR) rate of 33.3%; the median duration of response (DOR) and median progression-free survival (PFS) were not reached, with 90.6% of patients achieving tumor reduction and a median time to response (TTR) of 2.8 months (95% CI: 2.7-2.9 months). For follicular lymphoma (FL), the ORR was 45.3% (95% CI: 36.1%-54.8%) with a CR rate of 5.1% and PR rate of 40.2%; the median DOR was 11.1 months (95% CI: 8.3-15.6 months), median PFS was 10.6 months (95% CI: 7.2-13.7 months), 83.5% of patients had tumor reduction, and median TTR was 4.6 months (95% CI: 3.0-5.6 months). The overall ORR for MZL and FL was 47.1% in the intent-to-treat population, with 86.4% of all patients experiencing tumor volume reduction.	Common adverse reactions in greater than 20% of patients include diarrhea (59.1%), nausea (39.4%), fatigue (30.8%), vomiting (23.6%), and cough (20.7%). The most common grade 3–4 adverse reactions include neutropenia (11.5%) and diarrhea (10.1%). Grade ≥3 elevations in alanine aminotransferase (ALT) and aspartate aminotransferase (AST) occurred in 6.7% and 7.2% of patients, respectively. Serious treatment-emergent adverse events (TEAEs) were reported in 30.3% of patients, with the most frequent serious TEAEs (>1%) being diarrhea, acute kidney injury, anemia, dehydration, febrile neutropenia, pneumonia, sepsis, and urinary tract infection (1.4% each). Treatment-related TEAEs led to drug discontinuation in 14.9% of patients, with diarrhea (2.9%) and ALT/AST elevation (2.4%) being the most common grade ≥3 TEAEs causing discontinuation. Noninfectious colitis and pneumonitis occurred in 1.9% and 1.4% of patients, with grade ≥3 incidence of 0.5% and 1.0% respectively.	In 2022, the market was withdrawn	35
Inavolisib	PI3Kα	2024	Breast cancer associated with PIK3CA gene mutation	Adult patients diagnosed with locally advanced or metastatic breast cancer characterized by HR+, HER2− status, harboring PIK3CA mutations and exhibiting endocrine resistance	The median overall survival was 34.0 months (95% CI: 28.4 to 44.8) with inavolisib plus palbociclib–fulvestrant versus 27.0 months (95% CI: 22.8 to 38.7) with placebo plus palbociclib–fulvestrant (hazard ratio for death, 0.67; 95% CI: 0.48 to 0.94; P = 0.02). The median progression-free survival was 17.2 months with inavolisib combination therapy and 7.3 months with placebo combination therapy (hazard ratio for disease progression or death, 0.42; 95% CI: 0.32 to 0.55). The objective response rate was 62.7% (95% CI: 54.8 to 70.2%) in the inavolisib group versus 28.0% (95% CI: 21.3 to 35.6%) in the placebo group (P<0.001), with a median duration of response of 19.2 months and 11.1 months, respectively.	Common adverse reactions in greater than 20% of patients in the inavolisib group include hyperglycemia (63.4%), stomatitis or mucosal inflammation (55.3%), diarrhea (52.2%), thrombocytopenia (49.7%), anemia (39.8%), rash (26.7%), nausea (29.2%), and ocular toxic effects (29.2%). The most common grade 3–4 adverse reactions include neutropenia (82.6%), thrombocytopenia (13.7%), hyperglycemia (6.8%), and anemia (6.8%). Stomatitis or mucosal inflammation (5.6%), diarrhea (3.7%), and elevated AST or ALT level (4.3%) were also observed as grade 3–4 adverse events. Adverse events led to discontinuation of inavolisib in 6.8% of patients, and serious adverse events were reported in 27.3% of the inavolisib group.	Normally available for sale	30

FDA, Food and Drug Administration; CLL, chronic lymphocytic leukemia; FL, follicular lymphoma; SLL, small lymphocytic lymphoma; MZL, marginal zone lymphoma; HR+, hormone receptor−positive; HER2−, human epidermal growth factor receptor 2−negative.

To date, numerous clinical trials have evaluated the efficacy and safety of PI3K inhibitors in the treatment of breast cancer. The relevant data have been stored in public clinical trial databases and registration systems, such as the U.S. Clinical Trials Registry Platform, the Iranian Clinical Trials Registry Platform, the EU Clinical Trials Registration System, and the Chinese Clinical Trial Registry Center. However, some studies are still single-center, small-sample clinical trials, and their results are often inconsistent or even contradictory, making it difficult to provide comprehensive and reliable evidence for clinical practice. There is an urgent need to conduct a clinical trial landscape analysis based on large-scale data from multiple clinical trial databases and registration systems to clarify the efficacy and safety of PI3K inhibitors in the treatment of breast cancer.

In this context, this study aims to use data from eight authoritative public clinical trial databases and registration centers to conduct a systematic analysis of the role of PI3K inhibitors in the treatment of breast cancer. This study will systematically collect, screen, and analyze relevant clinical trial data to evaluate the clinical benefit rate (CBR), objective response rate (ORR), progression-free survival (PFS), overall survival (OS), and adverse events associated with PI3K inhibitors. Additionally, this study will conduct a systematic analysis of global clinical trial data on PI3K inhibitors for breast cancer to clarify the overall research trends, patterns of regional participation, and key efficacy and safety outcomes. It will also track shifting research priorities in clinical trials of PI3K inhibitors for the treatment of breast cancer and identify possible publication biases in the reporting of clinical trial results across different drug types, target sites, and trial stages, thereby clarifying the completeness and reliability of the current clinical evidence related to PI3K inhibitors for the treatment of breast cancer. Ultimately, it will provide an evidence-based reference for further optimizing clinical treatment strategies for breast cancer, guiding the subsequent research and development of PI3K inhibitors, and bridging the gap between clinical trial results and clinical application, thereby promoting the rational use and research advancement of PI3K inhibitors in the treatment of breast cancer.

## 2 Method

### 2.1 Identification and screening of the study

**Figure 1:**
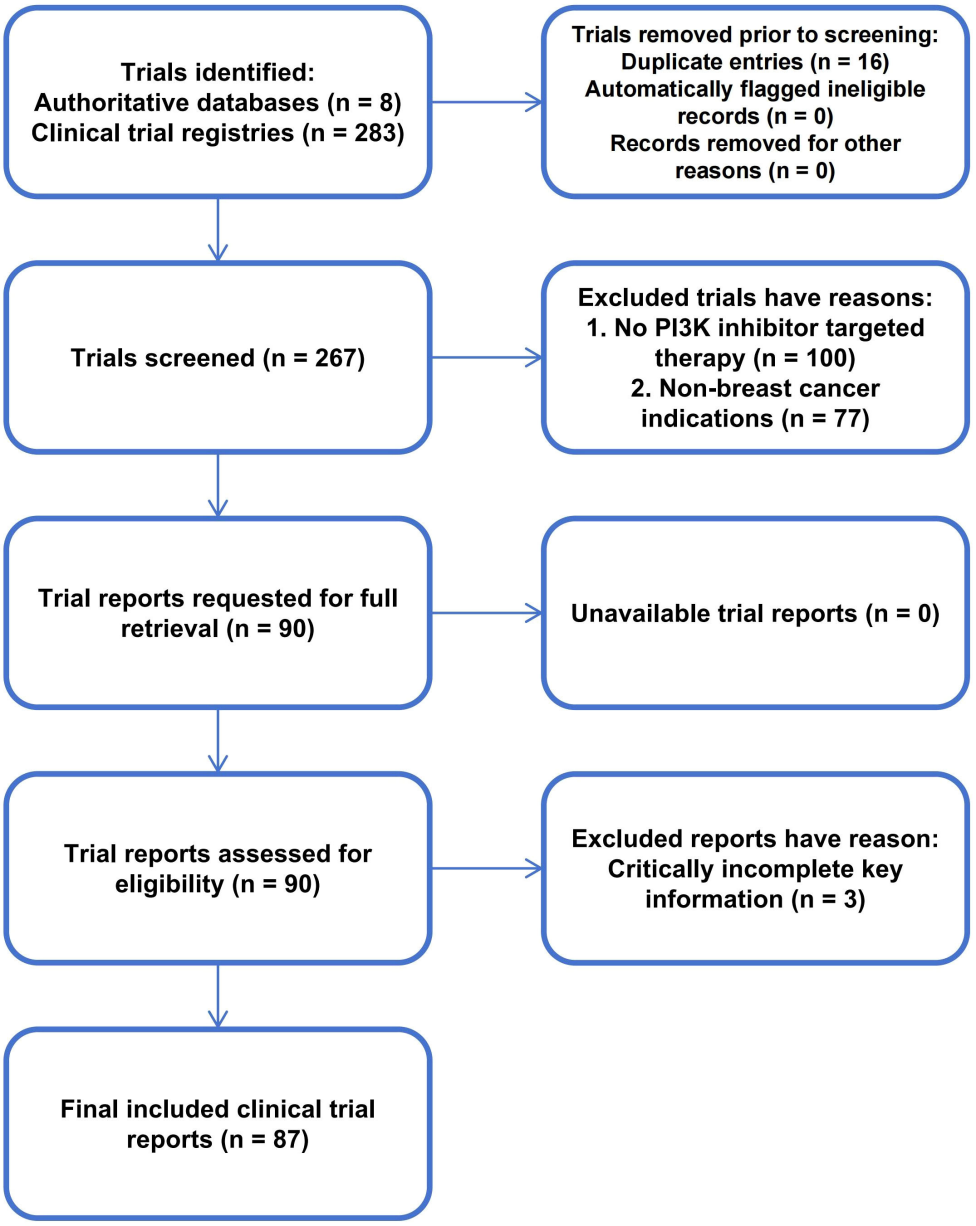
Clinical trial screening flowchart.

This study conducted a clinical trial landscape analysis of breast cancer-related clinical trials by searching eight major clinical trial registration databases (**Supplementary Appendix A1**). No restriction was placed on the start date, and the end date was set at January 1, 2026 (the date of the search). Using the control terms in PubMed MeSH and Embase Emtree systems, the search strategy included terms such as “Breast Cancer”, “Cancer of Breast”, “Breast Neoplasms”, “Phosphoinositide 3 Kinases”, and “Kinases, Phosphoinositide 3”, along with their synonyms and related terms. As an example, the complete search string was: (Breast Neoplasms OR Breast Neoplasm OR Neoplasm, Breast OR Neoplasms, Breast OR Breast Tumors OR Breast Tumor OR Tumor, Breast OR Tumors, Breast OR Breast Cancer OR Cancer, Breast OR Cancer of Breast OR Cancer of the Breast OR Malignant Neoplasm of Breast OR Breast Malignant Neoplasm OR Breast Malignant Neoplasms OR Malignant Tumor of Breast OR Breast Malignant Tumor OR Breast Malignant Tumors OR Mammary Cancer OR Cancer, Mammary OR Cancers, Mammary OR Mammary Cancers OR Mammary Neoplasms, Human OR Human Mammary Neoplasm OR Human Mammary Neoplasms OR Neoplasm, Human Mammary OR Neoplasms, Human Mammary OR Mammary Neoplasm, Human OR Breast Carcinoma OR Breast Carcinomas OR Carcinoma, Breast OR Carcinomas, Breast OR Mammary Carcinoma, Human OR Carcinoma, Human Mammary OR Carcinomas, Human Mammary OR Human Mammary Carcinomas OR Mammary Carcinomas, Human OR Human Mammary Carcinoma) AND (Phosphatidylinositol 3-Kinases OR Phosphatidylinositol 3 Kinases OR Phosphoinositide 3 Kinases OR Kinases, Phosphoinositide 3 OR PI3-Kinase OR PI3 Kinase OR Phosphatidylinositol-3-OH Kinase OR Kinase, Phosphatidylinositol-3-OH OR Phosphatidylinositol 3 OH Kinase OR Phosphoinositide 3-Hydroxykinase OR 3-Hydroxykinase, Phosphoinositide OR Phosphoinositide 3 Hydroxykinase OR PI 3-Kinase OR PI 3 Kinase OR PI-3 Kinase OR Kinase, PI-3 OR PtdIns 3-Kinase OR PtdIns 3 Kinase OR PI3 Kinases OR Kinases, PI3 OR PI-3K OR PtdIns 3-Kinases OR PtdIns 3 Kinases). The detailed search terms and records for each clinical trial database can be found in Appendix 1. A total of 283 potentially eligible studies were initially identified, and the screening process was conducted by researchers Cao and Chen (**Figure 1**). The specific process was as follows:

1. Repeated record elimination stage: By using an Excel management tool, the core information (such as trial registration number, research team, research institution, sample size, patient baseline characteristics, and research design) was compared to identify and delete duplicate records. If the core information was highly overlapping but the trial registration number was missing, the records were further determined to be duplicates through cross-validation of the study title and registration time.
2. Preliminary screening based on title, intervention measures and disease type: Firstly, conduct a preliminary screening based on the title, intervention measures and disease type to exclude studies that clearly do not meet the inclusion criteria. The inclusion criteria are as follows: (1) The indications are limited to breast cancer, or the research subjects must meet the standards of the American Society of Clinical Oncology/American Society of Pathology (ASCO/CAPs) (18–21); (2) The core assessment content must involve PI3K inhibitor targeted therapy, whether it is monotherapy or combination therapy. The PI3K inhibitors included in the search are as follows: Pictilisib, Buparlisib, Alpelisib, Dactolisib, Taselisib, Gedatolisib, Bimiralisib, etc. (covering approved and ongoing PI3K inhibitors targeting PI3Kα, PI3Kβ, PI3Kγ, PI3Kδ or multi-target combinations); (3) All trials that fully meet the above inclusion criteria are included in the analysis, regardless of their trial status (such as completed, recruiting, ongoing but not recruited, unknown status, etc.), whether the results have been published or not. The exclusion criteria are as follows: (1) The research topic is not related to breast cancer and the research subjects do not meet the ASCO/CAPs standards; (2) The guiding indications include not only breast cancer patients but also other cancer patients (such as non-small cell cancer, renal cell cancer); (3) Studies that do not involve PI3K targeted therapy and only evaluate other targets in the PI3K pathway (such as AKT inhibitors) or non-targeted drugs; (4) Important details of the clinical trial are missing even if supplemented by accessing the original trial registration information or contacting potential registrants (such as the name of the PI3K inhibitor, trial status, etc.).
3. Full-text screening stage: For the studies that pass the initial screening stage, all relevant information will be obtained and read to further verify whether they fully meet the inclusion criteria. The focus is on confirming the specific type of intervention measures (the type and dosage regimen of PI3K inhibitors), the purity of the study subjects’ indications (whether there are subgroups of non-breast cancer patients), and the completeness of the study design. Studies with incomplete information or hidden non-compliance with the inclusion criteria will be excluded.

During the screening process, two researchers (Cao and Chen) independently recorded the numbers of included and excluded trials at each stage, as well as the reasons for exclusion. Any disagreements arising during the screening process were first resolved through face-to-face discussion to reach a consensus. If no agreement was reached after discussion, a third researcher (**Song**) adjudicated the disagreement, and the arbitration result was regarded as the final screening decision.

### 2.2 Data extraction

The key elements extracted and compared included the year and phase of study initiation, molecular targets, trial type, trial status, sex and number of enrolled participants, drug name, and study region. Missing items in the trial registration records were supplemented by reviewing the original registration information or contacting the relevant registrants. Data extraction was completed by two researchers (Cao and Chen), and any inconsistencies or disputes were resolved through discussion with a third researcher (**Song**), who made the final decision.

### 2.3 Assessment of trial result disclosure status

We analyzed trials with a status of “Completed” and a completion time of ≥2 years. This analysis aimed to evaluate differences in trial result disclosure across different subgroups. The analyzed subcategories included: (1) Drug level: comparing the disclosure of trial results between approved mature drugs and the trial drugs; (2) Target level: comparing the disclosure of trial results for single-target (such as PI3Kα) and multi-target (such as PI3Kα/δ) PI3K inhibitors; (3) Trial stage level: comparing the disclosure of trial results for Phase 0, Phase I, Phase II, Phase III, Phase IV, and Phase II/III trials. The determination of the disclosure status was based on whether the database/registry retrieved contained publicly accessible primary or supplementary result data for this trial.

### 2.4 Statistical analysis

Statistical analysis mainly uses descriptive statistics. All statistical analyses are conducted using R 4.5.1 and SPSS 26.0. Frequency counts were performed for categorical variables, and the results were presented in numerical (percentage) form.

## 3 Results

A total of 87 trials were ultimately found to meet the inclusion criteria (Appendix 2). Cohen’s kappa coefficient was used to evaluate the inter-rater reliability of binary inclusion/exclusion judgments for 283 trials made by two independent reviewers (Cao and Chen). The observed agreement was 96.11% (272/283), and the Cohen’s kappa coefficient was 0.88, indicating almost perfect agreement beyond chance between the two reviewers. The statistical analysis showed that, except for one trial involving expanded access (NCT05134922), all remaining trials were interventional studies. A total of 36.7% (32/87) of the studies enrolled only female patients. More than half of the studies were collaborative trials (53.2%). Among these, NCT02437318 involved the largest number of participating countries, spanning 30 countries and 197 study centers. These findings indicate that collaborative trials occupy a dominant position in this research field, and that some key collaborative trials have established an international multicenter research pattern characterized by broad geographic coverage and numerous study centers.

### 3.1 International collaboration and population characteristics

**Figure 2:**
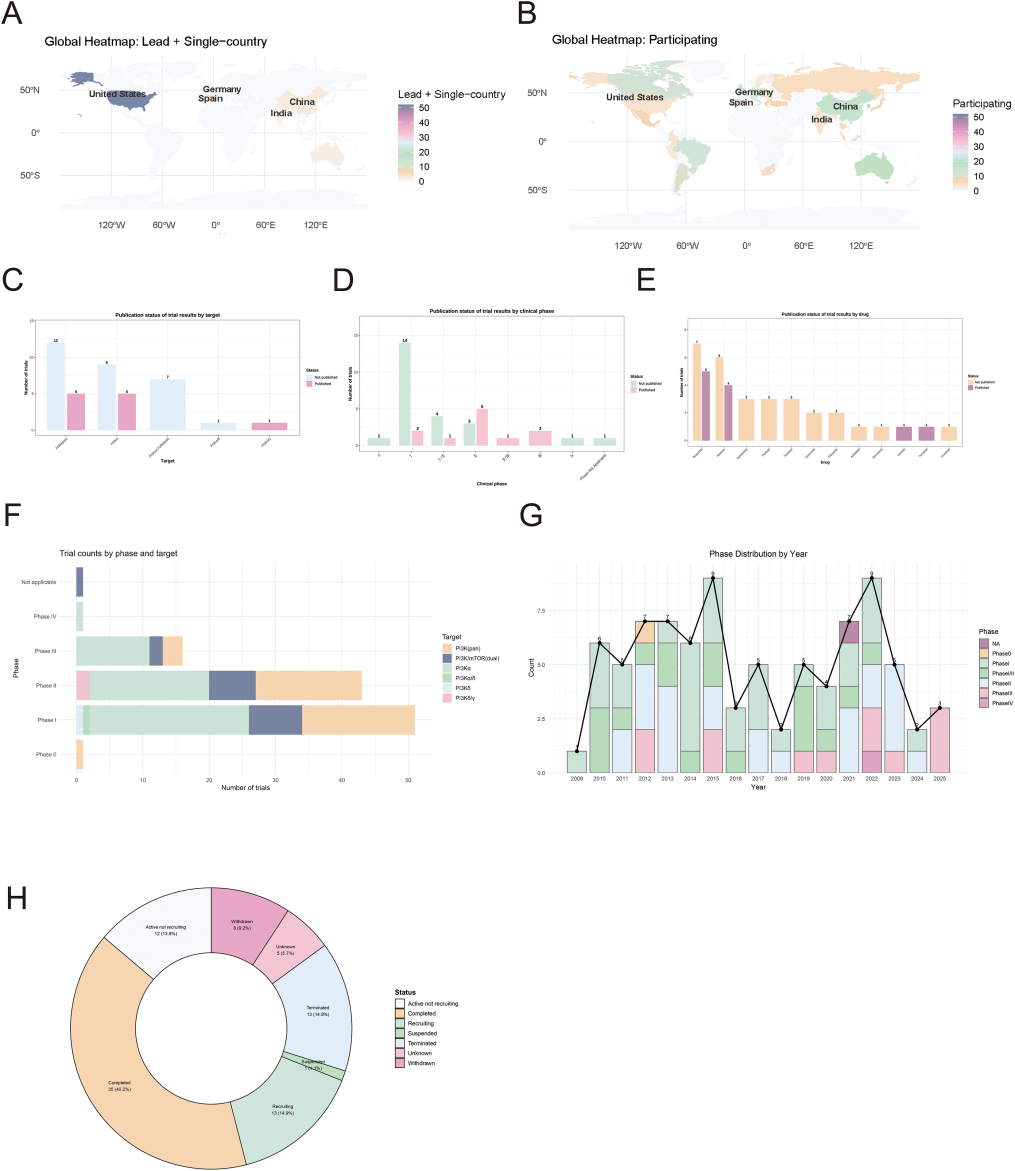
Comprehensive analysis of clinical trials of PI3K inhibitors used in the treatment of breast cancer. **(A)** World heat map of leading countries and individual countries; **(B)** World heat map of participating countries; **(C)** The result of publication bias in target points; **(D)** The results of publication bias during the trial phase; **(E)** The result of drug publication bias; **(F)** Analysis of stages and targets; **(G)** The statistical results of the experimental phases in different years; **(H)** State pie chart of the experiment.

The distribution of three core roles across countries—trial leadership, single-country conduct, and collaborative participation—reflects the allocation of global clinical trial resources and the heterogeneity of regional research capacity (**Figures 2A, B****;** **Table 2**, **3**). By total trial volume, the United States ranked first with 56 trials and held an unrivaled leading position. It led 31 trials, conducted 21 single-country trials, and participated in only four collaborative trials, indicating a model dominated by independent initiation and domestic validation. Spain ranked second with 37 trials and served as a pivotal Southern European hub. It led four trials, conducted three single-country trials, and participated in 30 collaborative trials, the highest number worldwide, serving as a core European research and coordination node. France ranked third with 26 trials and acted as a key European collaborator, with one led trial, two single-country trials, and 23 collaborative participations, reflecting a collaboration-centered pattern.

**Table 2:** Distribution of experiments across the country.

Country	Lead	Single-country	Participating	Total
United States	31	21	4	56
Spain	4	3	30	37
France	1	2	23	26
Italy	0	0	23	23
United Kingdom	1	0	22	23
Belgium	1	0	21	22
Australia	1	1	18	20
China	1	2	16	19
Germany	2	1	16	19
South Korea	0	0	19	19
Brazil	0	0	13	13
Canada	0	0	12	12
Hungary	0	0	11	11
Argentina	0	0	11	11
Poland	0	0	10	10
Israel	0	0	9	9
Austria	0	0	9	9
Czech Republic (Czechia)	0	0	9	9
Japan	0	0	8	8
Turkey	0	0	7	7
Mexico	0	0	7	7
Greece	0	0	7	7
Singapore	0	0	7	7
Netherlands	0	0	7	7
India	0	2	4	6
Thailand	0	0	6	6
Russia	0	0	5	5
Bulgaria	0	0	5	5
South Africa	0	0	5	5
Malaysia	0	0	5	5
Colombia	0	0	4	4
Romania	0	0	4	4
Sweden	0	0	3	3
Switzerland	0	0	3	3
Peru	0	0	3	3
Lebanon	0	0	3	3
Denmark	0	0	2	2
Luxembourg	0	0	2	2
Norway	0	0	2	2
Slovakia	0	0	2	2
Georgia	0	1	1	2
Finland	0	0	2	2
Portugal	0	0	2	2
Ukraine	0	0	1	1
Uganda	0	0	1	1
Croatia	0	0	1	1
Slovenia	0	0	1	1
New Zealand	0	0	1	1
Chile	0	0	1	1
Tunisia	0	0	1	1
Jordan	0	0	1	1
Kenya	0	0	1	1
Oman	0	0	1	1

**Table 3:** Distribution of experiments in the region.

Region	Lead	Participating	Single-country
North America	31	1	21
Europe	9	41	7
Asia–Pacific	2	38	5
Latin America & Caribbean	0	24	0
Other (Africa/Middle East)	0	19	0

1.North America: United States, Canada.

2.Europe: Spain, Poland, Hungary, Belgium, Germany, Russia, Czech Republic (Czechia), United Kingdom, Bulgaria, Slovakia, Ukraine, Italy, France, Serbia, Romania, Greece, Portugal, Switzerland, Ireland, Latvia, Belarus, Netherlands, Bosnia and Herzegovina, Austria, Estonia, Croatia, Lithuania, Sweden, Georgia, Denmark, Finland, Norway, Moldova (Republic of Moldova), Slovenia, Serbia and Montenegro, European Union (EU).

3.Asia–Pacific (Asia and Oceania): China, South Korea, Japan, Malaysia, Kazakhstan, India, Thailand, Singapore, Philippines, Bangladesh, Australia, New Zealand, North Korea.

4.Latin America and the Caribbean: Argentina, Brazil, Chile, Mexico, Colombia, Guatemala, Peru, Puerto Rico.

5.Other (Africa and Middle East, etc.): South Africa, Israel, Egypt, Tunisia, Réunion.

6.NCT01240928, NCT01288092, NCT01658176, NCT01953445, NCT03377101, NCT04142554, NCT04849364, NCT05967286, all three columns are empty and are not included in any region or column.

Italy and the United Kingdom tied for fourth, with 23 trials each, and Belgium ranked sixth with 22 trials; together, they formed the core collaborative force in Europe. Italy contributed exclusively through collaborative trials (22). The United Kingdom and Belgium each led one trial and participated in 22 and 21 collaborative trials, respectively, acting as coordination hubs in Western Europe. Australia ranked seventh with 20 trials and represented a balanced Oceanian hub, including one led trial, one single-country trial, and 18 collaborative participations. China, Germany, and South Korea tied for eighth, with 19 trials each. China led one trial, conducted two single-country trials, and participated in 16 collaborative trials. Germany led two trials, conducted one single-country trial, and participated in 16 collaborative trials. South Korea contributed exclusively through collaborative trials (4), highlighting its role as a core Asia-Pacific node.

A second echelon of countries participating exclusively in collaborative trials included Brazil (5), Canada (10), Hungary and Argentina (11 each), and Poland (23). These countries provided regional population data from Latin America, North America, and Central and Eastern Europe. Israel, Austria, and the Czech Republic (9 each), as well as Japan (14), Thailand (7), and another seven countries (7 trials each), further expanded the global network through collaborative participation. A small number of countries conducted localized single-country trials, including India (two single-country trials; four total trials) and Georgia (one single-country trial; two total trials), along with other low-volume contributors such as Romania, Sweden, Switzerland, and Peru. Despite their small trial numbers, these regions contributed to the ethnic, clinical, and geographic diversity of the global evidence base.

Together, these findings reveal a hierarchical global landscape: the United States dominates trial initiation and leadership; European countries form a dense multinational collaborative network, with Spain serving as a key regional initiator; and the Asia-Pacific region, Latin America, Central and Eastern Europe, and West Asia contribute research resources and diverse population data. This structure preserves the traditional technological and organizational advantages of the United States, integrates European collaborative resources, and extends coverage to diverse populations and healthcare systems through emerging economies and regional hubs. It provides robust cross-regional clinical evidence for the validation of efficacy and safety, thereby supporting the global translation and clinical confirmation of trial findings.

### 3.2 Assessment of trial result disclosure status

We assessed potential publication bias by examining the publication status of trial results from studies classified as “Completed” and with a duration of more than two years, stratified by therapeutic target, clinical phase, and individual drug (**Figures 2C–E**). Trials targeting PI3K(pan) were the most frequently included in the analysis (17 trials), and unpublished results (23) outnumbered published results (18). This was followed by trials targeting PI3K(α) (14 trials in total: 9 unpublished and 5 published) and PI3K/mTOR (dual) (7 trials in total, all unpublished). All other PI3K-related targets contributed only a limited number of trial results overall: PI3Kδ/γ had one published result, whereas PI3Kα/δ had one unpublished result. Across these therapeutic targets, unpublished trial results were more common than published results, with the greatest imbalance observed for PI3K(α), PI3K(pan), and PI3K/mTOR (dual). This trend suggests the possibility of publication bias, whereby positive findings for these key PI3K targets may be selectively reported, whereas negative or non-significant findings may remain undisclosed.

Phase I trials had the highest number of unpublished results (1), far exceeding all other phases, and only two published results were identified for this phase. Phase II was the only phase in which the number of published trials (18) exceeded the number of unpublished trials (24), and it also had the highest number of published results across all phases. For phase II/III and phase III trials, only published results were identified, with no unpublished data. By contrast, for phase 0, phase IV, and non-applicable studies, only unpublished results were observed. Phase I/II trials were predominantly unpublished. Overall, except for phases I and II, the total number of trials in each phase was low, and the numbers of published and unpublished results per phase were mostly between one and four.

At the individual drug level, some drugs were associated with a relatively large number of trial results, including both published and unpublished data. Most drugs, however, had only a limited number of trial results; some appeared to be dominated by published results, whereas others showed a mixed pattern of published and unpublished outcomes. These inter-drug differences suggest that the extent and pattern of potential publication bias may vary according to the specific therapeutic agent under investigation.

### 3.3 Stage and target analysis

The number of trials targeting components of the PI3K pathway was analyzed according to clinical phase and target subtype (**Figure 2F**). Among the 87 trials, the number of studies gradually decreased with advancing clinical development phases. Overall, phase I trials were the most common (n=30), followed by phase II (n=24) and phase III (n=12) trials. There were 17 phase I/II trials, one phase II/III trial, one phase 0 trial, and one phase IV trial. In addition, one trial was not assigned to a specific phase (“Not Applicable”).

When stratified by target subtype, PI3Kα was the most frequently investigated subtype across all phases, with most studies concentrated in the early stages of development. Specifically, 18 PI3Kα-targeting trials were conducted in phase I, 11 in phase II, and 9 in phase III, with additional studies conducted in the combined phase I/II and phase II/III categories. Trials targeting pan-PI3K (PI3K(pan)) and the dual PI3K/mTOR pathway were also prominent in early clinical development. There were 9 phase I and 7 phase II trials targeting PI3K(pan), as well as 5 phase I and 4 phase II trials targeting PI3K/mTOR (dual). This pattern reflects the continued exploration of broad-spectrum and combination inhibition strategies. In contrast, trials targeting more specific subtype combinations, such as PI3Kα/δ, PI3Kδ, and PI3Kδ/γ, were relatively limited and were observed mainly in the early phases. This suggests that these subtype-specific strategies remain at an early stage of clinical validation. The only phase IV trial targeted PI3Kα, indicating that this established target continues to attract attention even in later-stage development.

### 3.4 Analysis by year and stage

The temporal distribution of clinical trial phases from 2012 to 2025 was analyzed using a stacked bar chart, with an overlaid line indicating the total annual number of trials (**Figure 2G**). Over this 14-year period, the annual number of trials showed marked fluctuations, peaking at 9 in both 2015 and 2022 and reaching a low of 2 in 2018 and 2024. Across the remaining years, the annual count remained stable at 7 in 2012 and 2013, decreased to 6 in 2014, rebounded to the first peak in 2015, fluctuated between 3 and 5 from 2016 to 2020, rose to 7 again in 2021, and then declined to 5, 2, and 3 in 2023, 2024, and 2025, respectively, after the second peak in 2022.

The stacked bar chart further revealed distinct shifts in phase composition across periods. In the early period (2012–2015), trials were overwhelmingly concentrated in early-phase studies, with phase II and phase I/II trials being the most prevalent, along with a substantial number of phase I trials; no phase III or phase IV trials were recorded during this period. During the middle period (2016–2020), the overall trial volume declined. Phase I and phase I/II trials continued to dominate, whereas the proportion of phase II trials decreased. The trough in 2018 coincided with a narrow focus on phase I and phase II trials only, with late-phase trials remaining absent. In the late period (2021–2025), notable diversification in trial phases emerged alongside the 2022 peak in trial volume. Phase IV trials appeared for the first time in 2021 and 2022, while phase III trials emerged in 2022 and persisted through 2025, indicating a shift toward later-stage development. In addition, one “Not Applicable” trial was documented in 2021. Although the overall number of trials declined in the final three years, phase III trials remained a consistent component.

Overall, this analysis revealed two major patterns: a cyclical trial landscape, characterized by two distinct peaks and intervening troughs that may reflect periodic shifts in research investment or priorities, and a gradual maturation of the trial portfolio over time, as indicated by the increasing representation of later-phase trials in the latter years of the study period.

### 3.5 Analysis of test status

Among the 87 trials, those with a status of “Completed” accounted for the largest proportion (40.2%), indicating that nearly two-fifths of the trials had successfully concluded their planned protocols (**Figure 2H**). Trials with a status of “Recruiting” and “Terminated” shared the second-highest frequency (13 trials each, 14.9% each). The former represented studies that were still enrolling participants, whereas the latter referred to studies discontinued before completion. Trials with a status of “Active, not recruiting” (12 trials, 13.8%) referred to ongoing studies that were no longer enrolling new participants. Trials with a status of “Withdrawn” (8 trials, 9.0%), “Unknown status” (5 trials, 5.7%), and “Suspended” (1 trial, 1.1%) collectively accounted for the remaining 15.8% of the cohort, representing studies with interrupted, uncertain, or paused progress.

Overall, the status distribution showed that the largest proportion of included trials had been completed, whereas a notable proportion remained active (“Recruiting” plus “Active, not recruiting,” 28.7%). In addition, 25.0% of trials had been discontinued or suspended (“Terminated,” “Withdrawn,” and “Suspended”).

### 3.6 Efficacy and safety

This study provided a descriptive analysis of clinical trials investigating PI3K inhibitors for advanced breast cancer. All efficacy and safety data were derived from individual clinical trials(**Table 4**, **5**). Given the substantial heterogeneity across studies in terms of study design, enrolled populations, and treatment regimens, no direct cross-trial comparisons were performed, and no conclusions regarding comparative superiority were drawn.

**Table 4:** Clinical trial data of PI3K inhibitors in advanced breast cancer.

ID	Phase	Status	Population characteristics	Experimental group	PI3K target	Control group	PFS (experimental/control)	OS (experimental/control)	CBR (experimental/control)	ORR (experimental/control)	All-cause mortality (experimental/control)	Serious adverse events(SAEs)(≥grade 3) (experimental/control)	Common adverse events(SAEs)(experimental/control)
NCT01572727	Phase II/III	Completed	Patients with HER2-negative, locally advanced or metastatic breast cancer	Buparlisib 100 mg and hard gelatin capsules plus Paclitaxel	PI3K(pan)	Placebo plus Paclitaxel	8.0 (7.2-9.2)/9.2 (7.3-11.0)	29.5 (25.0-30.0)/NA^#^	26.2 (19.7-33.5)/32.9 (25.9-40.6)	22.6 (16.5-29.7)/27.1 (20.5-34.4)	--/--	61/202 (30.20%)/42/201 (20.90%)	196/202 (97.03%)/188/201 (93.53%)
NCT01610284	Phase III	Completed	Postmenopausal women with locally advanced or metastatic breast cancer (MBC) whose disease progressed during or after aromatase inhibitor (AI) treatment	Buparlisib 100mg + Fulvestrant	PI3K(pan)	Placebo + Fulvestrant	6.9 (6.8-7.8)/5.0 (4.0-5.2)	33.2 (30.0-37.3)/30.4 (27.9-32.2)	43.8 (39.7-47.9)/42.0 (37.9-46.2)	11.8 (9.3-14.7)/7.7 (5.7-10.2)	12/573 (2.09%)/13/570 (2.28%)	146/573 (25.48%)/101/570 (17.72%)	560/573 (97.73%)/485/570 (85.09%)
NCT02340221	Phase III	Terminated	Postmenopausal women with estrogen receptor-positive, HER2-negative, locally advanced or metastatic breast cancer who experienced disease recurrence or progression during or after aromatase inhibitor treatment	Taselisib + Fulvestrant 500 mg	PI3Kα	Placebo + Fulvestrant 500 mg	7.43 (7.26-9.07)/5.39 (3.68-7.29)	27.93 (24.38-NA^#^)/27.79 (24.25-33.35)	59.5 (53.6-65.4)/45.5 (37.2-54.3)	--/--	197/417 (47.24%)/100/214 (46.73%)	154/416 (37.02%)/23/213 (10.80%)	396/416 (95.19%)/183/213 (85.92%)
NCT02437318	Phase III	Completed	Men and postmenopausal women with HR-positive, HER2-negative advanced breast cancer whose disease progressed during or after AI treatment	Alpelisib 300mg + Fulvestrant 500 mg	PI3Kα	Placebo 300mg + Fulvestrant 500mg	11.0 (7.49-14.52)/5.7 (3.65-7.36)	39.3 (34.10-44.85)/31.4 (26.78-41.30)	61.5 (53.8-68.9)/44.8 (37.2-52.5)	26.6 (20.1-34.0)/13.4 (8.7-19.4)	9/284 (3.17%)/12/288 (4.17%)	110/284 (38.73%)/54/287 (18.82%)	280/284 (98.59%)/244/287 (85.02%)
NCT04191499	Phase I/II	Active not recruiting	Patients with PIK3CA-mutated, hormone receptor-positive, HER2-negative, locally advanced or metastatic breast cancer	Inavolisib 9mg + Palbociclib 125mg + Fulvestrant 500mg	PI3Kα	Placebo + Palbociclib 125mg + Fulvestrant 500mg	15 (11.3-20.5)/7.3 (5.6-9.3)	--/--	--/--	--/--	43/162 (26.54%)/54/162 (33.33%)	39/162 (24.07%)/17/162 (10.49%)	157/162 (96.91%)/158/162 (97.53%)
NCT01633060	Phase III	Terminated	Patients with hormone receptor-positive (HR-positive), human epidermal growth factor receptor 2-negative (HER2-negative), locally advanced or metastatic breast cancer treated with aromatase inhibitor (AI)	Buparlisib 100mg + Fulvestrant 500mg	PI3K(pan)	Placebo + Fulvestrant 500mg	3.9 (2.8-4.2)/1.8 (1.5-2.8)	21.2 (18.2-23.4)/22.1 (17.3-NA^#^)	--/--	--/--	123/288 (42.71%)/68/140 (48.57%)	74/288 (25.69%)/26/140 (18.57%)	271/288 (94.10%)/113/140 (80.71%)

#Upper limit of confidence interval was not estimable due to the low number of participants with events.

PFS, Progression-Free Survival; OS, Overall Survival; CBR, Clinical Benefit Rate; ORR, Objective Response Rate; SAEs, Serious Adverse Events; CAEs, Common Adverse Events.

**Table 5:** Data on adverse events of PI3K inhibitors.

ID	Phases	Interventions	FDA-approved indications	Target	SAEs(affected/at risk (%))	CAEs(affected/at risk (%))
NCT04191499	Phase I/II	Inavolisib	PIK3CA-mutated breast cancer	PI3Kα	COVID-19 (2.47%); Anemia (1.85%); Febrile neutropenia (1.85%); Pneumonia (1.85%); Acute coronary syndrome (1.23%); Diarrhea (1.23%); Pyrexia (1.23%); Urinary tract infection (1.23%); Thrombocytopenia (0.62%); Abdominal pain (0.62%); Anal fistula (0.62%); Gastrointestinal hemorrhage (0.62%); Intestinal perforation (0.62%); Nausea (0.62%); Vomiting (0.62%); Death (0.62%); Malaise (0.62%); Anal abscess (0.62%); Cellulitis (0.62%); Device-related infection (0.62%); Lung abscess (0.62%); Upper respiratory tract infection (0.62%); Ankle fracture (0.62%); Post-procedural fistula (0.62%); Procedural headache (0.62%); Increased alanine aminotransferase (0.62%); Increased aspartate aminotransferase (0.62%); Hypocalcemia (0.62%); Hypokalemia (0.62%); Tetany (0.62%); Flank pain (0.62%); Osteonecrosis of the jaw (0.62%); Cerebral hemorrhage (0.62%); Cerebrovascular accident (0.62%); Intracranial hemorrhage (0.62%); Syncope (0.62%); Acute kidney injury (0.62%); Dyspnea (0.62%); Skin ulcer (0.62%); Embolism (0.62%)	Neutropenia (54.32%); Hyperglycemia (53.70%); Diarrhea (48.15%); Decreased neutrophil count (38.89%); Anemia (35.80%); Stomatitis (32.72%); Nausea (27.16%); Decreased platelet count (25.93%); Fatigue (23.46%); Decreased appetite (23.46%); Thrombocytopenia (22.22%); Decreased white blood cell count (21.60%); COVID-19 (20.99%); Headache (20.99%); Mucosal inflammation (18.52%); Alopecia (18.52%); Leukopenia (17.28%); Increased alanine aminotransferase (17.28%); Increased aspartate aminotransferase (17.28%); Weight loss (17.28%); Rash (16.05%); Asthenia (15.43%); Hypokalemia (15.43%); Constipation (14.81%); Vomiting (14.81%); Urinary tract infection (11.73%); Back pain (11.11%); Cough (11.11%); Dry skin (11.11%); Abdominal pain (9.88%); Arthralgia (9.88%); Muscle spasms (9.88%); Insomnia (9.88%); Dry eye (8.64%); Upper abdominal pain (8.64%); Dyspepsia (8.02%); Gastroesophageal reflux disease (8.02%); Pyrexia (8.02%); Upper respiratory tract infection (8.02%); Increased glycosylated hemoglobin (8.02%); Hypocalcemia (8.02%); Dysgeusia (8.02%); Pruritus (7.41%); Dyspnea (6.79%); Increased blood insulin (6.17%); Dizziness (6.17%); Dry mouth (5.56%); Toothache (5.56%); Increased blood creatinine (5.56%); Extremity pain (5.56%); Oropharyngeal pain (5.56%); Decreased lymphocyte count (3.09%)
NCT04345913	Phase I/II	Copanlisib	Recurrent follicular lymphoma	PI3K (pan)	Non-cardiac chest pain (16.67%); Prolonged activated partial thromboplastin time (16.67%); Dyspnea (16.67%); Thromboembolic event (16.67%)	Decreased lymphocyte count (66.67%); Decreased white blood cell count (66.67%); Hyperglycemia (66.67%); Cough (66.67%); Anemia (50.00%); Fatigue (50.00%); Decreased neutrophil count (50.00%); Maculopapular rash (50.00%); Hypokalemia (33.33%); Hypomagnesemia (33.33%); Pruritus (33.33%); Radiation fibrosis (33.33%); Eosinophilia (16.67%); Otic fluid (16.67%); Periorbital edema (16.67%); Right eye pruritus/discharge (16.67%); Constipation (16.67%); Diarrhea (16.67%); Gastroesophageal reflux disease (16.67%); Hemorrhoidal hemorrhage (16.67%); Nausea (16.67%); Rectal hemorrhage (16.67%); Chills (16.67%); Flu-like symptoms (16.67%); Sinusitis (16.67%); Bruising (16.67%); Wound complication (16.67%); Increased alanine aminotransferase (16.67%); Increased alkaline phosphatase (16.67%); Increased cardiac troponin T (16.67%); Hypercholesterolemia (16.67%); Increased lymphocyte count (16.67%); Decreased platelet count (16.67%); Weight loss (16.67%); Anorexia (16.67%); Arthralgia (16.67%); Back pain (16.67%); Chest wall pain (16.67%); Generalized muscle weakness (16.67%); Myalgia (16.67%); Dysgeusia (16.67%); Headache (16.67%); Peripheral motor neuropathy (16.67%); Peripheral sensory neuropathy (16.67%); Breast pain (16.67%); Laryngeal hemorrhage (16.67%); Pneumonitis (16.67%); Intermittent pruritus (16.67%); Acneiform rash (16.67%); Skin lesion (16.67%); Hypertension (16.67%)
NCT02437318	Phase III	Alpelisib	PIK3CA-mutated breast cancer	PI3Kα	Hyperglycemia (9.86%); Diarrhea (3.17%); Osteonecrosis of the jaw (3.17%); Anemia (2.11%); Abdominal pain (2.11%); Acute kidney injury (2.11%); Nausea (1.76%); Vomiting (1.76%); Rash (1.76%); Stomatitis (1.41%); Pyrexia (1.41%); Dyspnea (1.41%); Mucosal inflammation (1.06%); Hypersensitivity (1.06%); Pneumonia (1.06%); Decreased appetite (1.06%); Dehydration (1.06%); Hypokalemia (1.06%); Supraventricular tachycardia (0.70%); Colitis (0.70%); Upper gastrointestinal hemorrhage (0.70%); General physical health deterioration (0.70%); Febrile neutropenia (0.35%); Thrombotic microangiopathy (0.35%); Coronary arteriospasm (0.35%); Atrial fibrillation (0.35%); Bradycardia (0.35%); Cardiac arrest (0.35%); Dyspepsia (0.35%); Enterocolitis (0.35%); Ileus (0.35%); Intestinal obstruction (0.35%); Large intestinal perforation (0.35%); Melena (0.35%); Esophagitis (0.35%); Pancreatitis (0.35%); Adverse drug reaction (0.35%); Asthenia (0.35%); Chest discomfort (0.35%); Chest pain (0.35%); Fatigue (0.35%); Malaise (0.35%); Multiple organ dysfunction syndrome (0.35%); Hepatic failure (0.35%); Acute hepatitis (0.35%); Anaphylactic reaction (0.35%)	Hyperglycemia (64.08%); Diarrhea (58.80%); Nausea (46.83%); Decreased appetite (36.27%); Rash (35.56%); Vomiting (28.17%); Weight loss (27.82%); Fatigue (25.70%); Stomatitis (24.65%); Asthenia (22.54%); Alopecia (20.42%); Pruritus (19.01%); Mucosal inflammation (18.66%); Headache (18.66%); Pyrexia (16.55%); Peripheral edema (16.20%); Arthralgia (15.49%); Back pain (15.49%); Dry skin (15.49%); Dysgeusia (13.73%); Increased blood creatinine (12.68%); Cough (12.68%); Abdominal pain (11.62%); Dyspepsia (11.27%); Increased aspartate aminotransferase (11.27%); Dry mouth (10.56%); Anemia (10.21%); Extremity pain (10.21%); Hypertension (10.21%); Constipation (8.80%); Upper abdominal pain (6.69%); Blurred vision (5.28%)
NCT02340221	Phase III	Taselisib	None	PI3Kα	Nausea (0.94%); Pyrexia (0.94%); Cerebrovascular accident (0.94%); Pleural effusion (0.94%); Anemia (0.47%); Vomiting (0.47%); Chills (0.47%); Death (0.47%); Fatigue (0.47%); Pain (0.47%); Facial swelling (0.47%); Pneumonia (0.47%); Sepsis (0.47%); Fall (0.47%); Femoral fracture (0.47%); Cervical fracture (0.47%); Hip fracture (0.47%); Wrist fracture (0.47%); Increased alanine aminotransferase (0.47%); Increased aspartate aminotransferase (0.47%); Decreased white blood cell count (0.47%); Dehydration (0.47%); Muscle spasms (0.47%); Muscular weakness (0.47%); Osteonecrosis of the jaw (0.47%); Facial paralysis (0.47%); Seventh cranial nerve paralysis (0.47%); Ureterolithiasis (0.47%); Dyspnea (0.47%); Pleuritic pain (0.47%); Pneumonitis (0.47%); Pulmonary edema (0.47%)	Nausea (25.35%); Diarrhea (21.13%); Asthenia (18.78%); Fatigue (18.78%); Arthralgia (16.43%); Cough (15.96%); Constipation (15.02%); Back pain (13.15%); Headache (12.68%); Hot flush (12.68%); Vomiting (11.74%); Decreased appetite (10.80%); Hyperglycemia (9.86%); Anemia (9.39%); Abdominal pain (9.39%); Extremity pain (8.92%); Dry mouth (8.45%); Dizziness (8.45%); Insomnia (8.45%); Pruritus (8.45%); Bone pain (7.98%); Dyspnea (7.98%); Rash (7.98%); Increased aspartate aminotransferase (7.04%); Myalgia (6.57%); Mucosal inflammation (5.16%); Peripheral edema (5.16%); Upper respiratory tract infection (5.16%); Hypertension (5.16%); Increased blood alkaline phosphatase (5.63%); Neutropenia (4.23%); Pyrexia (4.23%); Urinary tract infection (4.23%); Increased alanine aminotransferase (4.23%); Dry skin (4.69%); Upper abdominal pain (3.76%); Stomatitis (3.76%); Increased blood creatinine (2.82%); Weight loss (2.82%); Muscle spasms (2.82%); Alopecia (2.82%); Dyspepsia (2.35%); Dysgeusia (2.35%); Gastroesophageal reflux disease (1.88%); Hypokalemia (0.94%)
NCT01572727	Phase II/III	Buparlisib	None	PI3K (pan)	Pyrexia (3.47%); Pneumonitis (2.97%); Diarrhea (2.48%); Urinary tract infection (1.98%); Asthenia (1.49%); Sepsis (1.49%); Deep vein thrombosis (1.49%); Neutropenia (0.99%); Vomiting (0.99%); Device-related infection (0.99%); Fall (0.99%); Hyperglycemia (0.99%); Confusional state (0.99%); Mental disorder (0.99%); Mental status changes (0.99%); Interstitial lung disease (0.99%); Rash (0.99%); Skin ulcer (0.99%); Hypertension (0.99%); Pericardial effusion (0.50%); Cataract (0.50%); Nausea (0.50%); Herpes zoster (0.50%); Head injury (0.50%); Asthma (0.50%)	Diarrhea (53.96%); Alopecia (50.99%); Rash (42.57%); Nausea (41.09%); Hyperglycemia (40.59%); Fatigue (33.17%); Neutropenia (31.68%); Decreased appetite (31.68%); Stomatitis (27.72%); Peripheral neuropathy (24.75%); Asthenia (23.76%); Constipation (23.27%); Anemia (22.77%); Cough (19.80%); Vomiting (19.31%); Dysgeusia (19.31%); Increased alanine aminotransferase (17.33%); Headache (17.33%); Dizziness (16.34%); Peripheral sensory neuropathy (15.35%); Increased aspartate aminotransferase (14.85%); Weight loss (14.85%); Pyrexia (14.36%); Abdominal pain (13.37%); Extremity pain (13.37%); Dyspepsia (11.39%); Arthralgia (11.39%); Blurred vision (9.41%); Hypertension (8.42%)

SAEs, Serious Adverse Events; CAEs, Common Adverse Events.

Due to the limited disclosure of detailed adverse event classification data in some included trials (e.g., incomplete reporting of CAE subtypes or lack of stratified SAE data), it is not feasible to extend **Table 5** to all 87 trials.

Among the six included breast cancer trials, NCT01572727 (phase II/III; HER2-negative advanced breast cancer) evaluated buparlisib plus paclitaxel. The median PFS was 8.0 (7.2–9.2) months in the experimental group and 9.2 (7.3–11.0) months in the control group. The incidences of grade ≥3 SAEs and CAEs were 30.20% and 97.03%, respectively, in the experimental group, compared with 20.90% and 93.53% in the control group. NCT01610284 (phase III; postmenopausal advanced breast cancer progressing on AI therapy) evaluated buparlisib plus fulvestrant. The median PFS was 6.9 (6.8–7.8) months versus 5.0 (4.0–5.2) months. Grade ≥3 SAEs and CAEs occurred in 25.48% and 97.73% of patients in the experimental group, respectively, versus 17.72% and 85.09% in the control group. NCT02340221 (phase III; ER+/HER2− advanced breast cancer progressing on AI therapy) assessed taselisib plus fulvestrant. The median PFS was 7.43 (7.26–9.07) months versus 5.39 (3.68–7.29) months. Grade ≥3 SAEs and CAEs were reported in 37.02% and 95.19% of patients in the experimental group, respectively, compared with 10.80% and 85.92% in the control group. NCT02437318 (phase III; HR+/HER2− advanced breast cancer progressing on AI therapy) evaluated alpelisib plus fulvestrant. The median PFS was 11.0 (7.49–14.52) months versus 5.7 (3.65–7.36) months. Grade ≥3 SAEs and CAEs occurred in 38.73% and 98.59% of patients in the experimental group, respectively, versus 18.82% and 85.02% in the control group. NCT04191499 (phase I/II; PIK3CA-mutated HR+/HER2− advanced breast cancer) evaluated inavolisib plus palbociclib and fulvestrant. The median PFS was 15.0 (11.3–20.5) months versus 7.3 (5.6–9.3) months. Grade ≥3 SAEs and CAEs were reported in 24.07% and 96.91% of patients in the experimental group, respectively, compared with 10.49% and 97.53% in the control group. NCT01633060 (phase III; HR+/HER2− advanced breast cancer after AI therapy) evaluated buparlisib plus fulvestrant. The median PFS was 3.9 (2.8–4.2) months versus 1.8 (1.5–2.8) months. Grade ≥3 SAEs and CAEs occurred in 25.69% and 94.10% of patients in the experimental group, respectively, versus 18.57% and 80.71% in the control group.

Notably, OS, CBR, ORR, and all-cause mortality were not reported in some of the above trials. In addition, in the phase I/II trial NCT04345913, no breast cancer-related efficacy data were available for copanlisib, which is approved for relapsed follicular lymphoma. The most common SAEs were non-cardiac chest pain and thromboembolic events (16.67% each), whereas the most common CAEs included decreased lymphocyte and leukocyte counts and hyperglycemia (66.67% each).

## 4 Discussion

This study systematically analyzed the current clinical research status of PI3K inhibitors in breast cancer treatment. Based on 8 authoritative clinical trial databases and registration systems, 87 trials that met the inclusion criteria were screened, leading to several key findings: Firstly, the distribution characteristics of the research stages were significant. The proportion of phase I clinical trials was the highest (34.5%, n=30), followed by phase I/phase II combined trials (19.5%, n=17). There were 24 phase II and 12 phase III trials, with only 1 phase IV trial. This indicates that the research on breast cancer PI3K inhibitors is still centered on early dose exploration and mid-term efficacy confirmation, while late confirmatory trials are gradually advancing. Secondly, the targets and drug development were highly concentrated. PI3Kα was the core target (46 related trials), far exceeding pan-PI3K (28 trials) and the dual-target PI3K/mTOR (15 trials). Alpelisib, as the first approved breast cancer PI3K inhibitor, covered the entire development stage from phase I to phase IV, being a benchmark drug in this field. Furthermore, the research mode presented a trend of mainly combined therapy and significant international collaboration. More than half of the studies were combined trials (53.2%). The United States led 56 trials as the global research core, Europe formed a dense transnational collaboration network, and emerging economies such as China participated deeply. At the same time, this study found that there was a significant publication bias in this field, which became a key issue affecting the completeness and reliability of clinical evidence, and was also one of the important core findings of this analysis.

This study systematically analyzed the current clinical research landscape of PI3K inhibitors in breast cancer treatment. Based on eight authoritative clinical trial databases and registration systems, 87 trials meeting the inclusion criteria were identified, yielding several key conclusions. First, the distribution of clinical development phases showed distinct characteristics. Phase I trials accounted for the largest proportion (34.5%, n=30), followed by phase I/II trials (19.5%, n=17). There were 24 phase II trials and 12 phase III trials, whereas only one phase IV trial was identified. These findings suggest that clinical research on PI3K inhibitors for breast cancer remains centered on early dose exploration and mid-stage efficacy evaluation, whereas late-stage confirmatory trials are gradually emerging.

Second, research into therapeutic targets and drug development for PI3K inhibitors in breast cancer was highly concentrated around specific subtypes and agents, with a clear focus on core targets and benchmark drugs. PI3Kα was the dominant target (46 related trials), far exceeding pan-PI3K (28 trials) and dual PI3K/mTOR targeting (15 trials). As the first PI3K inhibitor approved for breast cancer, alpelisib spanned the full clinical development spectrum from phase I to phase IV and has become a benchmark drug in this field. Furthermore, the overall research pattern was characterized by combination therapy and extensive international collaboration. More than half of the studies were collaborative trials (53.2%). The United States led 56 trials and served as the global research hub, Europe formed a dense transnational collaborative network, and emerging economies such as China were deeply involved in this field. At the same time, this study identified potential publication bias in this field, which may represent a key issue affecting the completeness and reliability of the clinical evidence base and constitutes an important finding of the present analysis.

This analysis of publication and reporting status, stratified by therapeutic target, clinical phase, and drug type, revealed substantial differences across all dimensions. At the target level, more than 60% of trials involving the core targets remained unpublished, with unreported rates of 64.29% for PI3Kα and 70.58% for pan-PI3K, while no results were reported for dual PI3K/mTOR-targeting trials. At the clinical phase level, phase I trials showed particularly limited disclosure, with only two reported results, whereas phase II was the only phase in which published results outnumbered unpublished results. At the drug level, approved PI3Kα inhibitors were associated with more complete result disclosure, while several unapproved agents showed limited public reporting. Together, these findings suggest the presence of potential reporting bias, whereby negative or clinically non-beneficial results may be underreported.

Among PI3K inhibitors investigated for breast cancer, only alpelisib (approved in 2019) (7) and inavolisib (approved in 2024) (25) have so far received FDA approval for PIK3CA-mutated HR+/HER2− advanced disease. The remaining PI3K inhibitors have not received approval for breast cancer-related indications. From the perspective of clinical efficacy, alpelisib combined with fulvestrant achieved a median PFS of 11.0 months, compared with 5.7 months in the control group, and a median OS of 39.3 months, compared with 31.4 months in the control group, in the NCT02437318 trial. This makes alpelisib the only PI3K inhibitor that has demonstrated an OS benefit in a breast cancer trial to date. Inavolisib combined with palbociclib and fulvestrant achieved a median PFS of 15.0 months, suggesting a promising new strategy for combination treatment in this subtype.

In contrast, the pan-PI3K inhibitor buparlisib showed only a modest PFS benefit in AI-resistant HR+/HER2− breast cancer, without an associated OS benefit, and its combination with paclitaxel failed to demonstrate a therapeutic advantage. Taselisib showed slight improvements in PFS and CBR, but these findings were insufficient to support regulatory approval for clinical use in breast cancer. However, potential publication bias may have introduced important distortions into the current clinical understanding of the efficacy and safety of PI3K inhibitors. Unpublished negative findings cannot be systematically integrated, which may lead to overestimation of treatment benefits, while the safety profiles of some agents cannot be fully evaluated because of incomplete public reporting. This also makes it difficult to examine key issues in depth, including the molecular basis of trial failure and potential biases in population selection. As a result, the optimization of PI3K inhibitor development may be hindered, and the formulation of individualized treatment strategies in clinical practice becomes more challenging.

This study included 87 clinical trials spanning multiple regions and populations worldwide, thereby providing multidimensional clinical evidence for the application of PI3K inhibitors in breast cancer. The efficacy of PI3Kα inhibitors in PIK3CA-mutated HR+/HER2− advanced breast cancer has been well established, suggesting that survival outcomes in this subtype may be improved and that breast cancer treatment is moving toward more precise target selection, individualized therapy, and optimization of combination regimens. However, potential publication bias has substantially compromised the completeness of the available clinical evidence, and the target-specific adverse event profile of PI3K inhibitors makes their clinical use highly dependent on PIK3CA testing results (26). Therefore, in addition to providing specialized training for clinicians on PI3K pathway testing, treatment indications, and adverse event management, addressing publication bias and improving the clinical evidence system should be regarded as urgent priorities.

Future research needs to promote the optimized application of PI3K inhibitors in breast cancer treatment from multiple perspectives. Addressing publication bias specifically is the fundamental prerequisite. The core exploration directions are as follows: Firstly, strengthen the mandatory public disclosure and complete reporting system for clinical trial results. Regulatory agencies should clearly require that all PI3K inhibitor clinical trials registered on authoritative platforms, regardless of whether the results are positive, negative, or have no clear conclusion, must disclose the main and secondary outcome data on time. Establish a unified global clinical trial data sharing platform to break down data barriers and enhance the research transparency in the entire field. At the same time, encourage pharmaceutical companies and research teams to disclose the detailed research data of negative trials, including population stratification, medication regimens, failure reasons, etc., to provide real and comprehensive references for subsequent research to avoid duplicate studies and optimize research directions. Secondly, deepen research into PI3K pathway-related biomarkers to develop more precise and predictive stratification tools for breast cancer patients receiving PI3K inhibitor therapy. Develop multi-dimensional biomarker combinations based on indicators such as PIK3CA mutation subtypes and PTEN expression status to further optimize patient stratification. The conduct of this research requires unbiased and complete clinical trial data to ensure the accuracy and applicability of biomarkers (22, 27). Again, conduct in-depth research on the resistance and safety issues of PI3K inhibitors, deeply explore the resistance mechanisms such as FGFR1 overexpression and reactivation of the PI3K/AKT/mTOR pathway, and develop combined anti-resistance regimens using PI3Kα inhibitors in combination with FGFR and mTOR inhibitors; at the same time, proactively address the target-specific toxicities linked to PI3K inhibitors—including hyperglycemia and osteonecrosis of the jaw with PI3Kα inhibitors (28), and infections and cardiovascular adverse events with pan-PI3K inhibitors (23), by developing individualized adverse reaction management strategies and optimizing drug molecular structures to enhance target selectivity and reduce off-target toxicity. However, to translate these clinical insights into mechanistic understanding, appropriate preclinical models of cancer and its associated complications are required (29). Additionally, the application scope of PI3K inhibitors should be expanded by investigating their role in breast cancer subtypes such as triple-negative breast cancer and HER2-low breast cancer. Large-scale phase III clinical trials are also needed to verify the long-term efficacy of combination regimens involving PI3Kα inhibitors with CDK4/6 inhibitors, antibody–drug conjugates (ADCs), and other agents. The design and implementation of these studies should also address previous publication bias to avoid potential design flaws arising from a distorted evidence base. Finally, in response to regional disparities in the global clinical trial landscape of PI3K inhibitors, international collaboration and clinical research capacity building in emerging economies should be strengthened. Multicenter and multi-population clinical trials should be encouraged, and complete reporting of trial results should be ensured, thereby providing more comprehensive population-based evidence for the global application of PI3K inhibitors.

In summary, PI3K inhibitors have made substantial progress in the treatment of breast cancer, particularly in PIK3CA-mutated HR+/HER2− advanced breast cancer. The approval of alpelisib and inavolisib has provided new targeted treatment options for this patient population and established these agents as important components of comprehensive treatment strategies for this breast cancer subtype. However, significant publication bias in this field, together with drug resistance, target-specific safety concerns, a limited scope of application, and regional disparities in the global research landscape, has substantially constrained the broader clinical application of PI3K inhibitors. With improvements in mandatory clinical trial data disclosure, the gradual correction of publication bias, and continued advances in precision medicine, the clinical use of PI3K inhibitors is expected to become more precise and standardized, while the supporting evidence base will become more complete and reliable. These advances may ultimately bring survival benefits to a broader population of patients with breast cancer and promote the further refinement of targeted therapy in this field.

Although this study reviewed the clinical trial landscape of PI3K inhibitors in the treatment of breast cancer and clearly presented the evolutionary characteristics of research phases, regional participation patterns, and differences in efficacy and safety among various PI3K inhibitor targets, thereby providing an evidence-based reference for the clinical practice of targeted therapy in breast cancer and for future research on PI3K inhibitors, several limitations should be acknowledged. First, publication bias may have resulted in the available clinical evidence being disproportionately concentrated on approved and more mature drugs, particularly PI3Kα-targeted agents and phase III trials. Second, this study did not incorporate a systematic framework for assessing clinical trial quality, nor did it perform subgroup-stratified analyses to account for global geographic heterogeneity. Third, due to the limited accessibility of publicly available registration data, we were unable to investigate in depth the specific internal reasons underlying unpublished trials, nor could we consistently extract detailed information on trial termination or withdrawal from the original trial registry records. In addition, substantial heterogeneity existed among the included studies, making reliable direct comparisons of efficacy across trials infeasible. Therefore, the data presented in this article should be interpreted as a comprehensive summary of efficacy outcomes from publicly available clinical trials rather than as a basis for direct comparisons among different treatment strategies. Furthermore, mature data on key endpoints such as OS and ORR for trials including NCT04191499 are not yet publicly available. The absence of these core efficacy endpoints reduces the completeness of the available efficacy data and limits our ability to comprehensively integrate and comparatively evaluate the activity of different treatment regimens. Collectively, these limitations may have affected the completeness, credibility, and regional generalizability of the study conclusions.

## 5 Conclusion

In summary, analysis of 87 clinical trials involving 87 PI3K inhibitors for breast cancer in eight major clinical databases indicates that selective inhibitors targeting PI3Kα(Alpelisib and Inavolisib) are the most clinically promising drugs for HR+/HER2− advanced breast cancer with PIK3CA mutations, especially when used in combination with endocrine therapy. Aplicib (in the Phase III trial NCT02437318) doubled the median progression-free survival (PFS) and achieved the only overall survival (OS) benefit among all the PI3K inhibitors in the studies, while inavilosib (in the I/II phase trial NCT04191499), when used in combination with palbociclib and fulvestrant, the median PFS reached 15.0 months - more than twice that of the control group. In contrast, the efficacy of pan-PI3K and dual PI3K/mTOR inhibitors is limited and more toxic. The high rate of severe non-publication (over 60% of key target trials were not published), drug resistance, target-specific toxicity, and regional research differences remain major obstacles in clinical application. Future research should focus on mandatory reporting of trial results, patient stratification based on biomarkers, optimization of combination treatment regimens, and international cooperation to improve the accuracy and accessibility of breast cancer treatment regimens based on PI3K inhibitors.

## Data Availability

The original contributions presented in the study are included in the article/**Supplementary Material**. Further inquiries can be directed to the corresponding author.
