# Supplementary material for: Application of PI3K inhibitors in breast cancer treatment: a clinical trial landscape analysis based on clinical trial databases and registries
Source: Front Oncol. 2026 Apr 13;16:1807951. doi: 10.3389/fonc.2026.1807951 (PMC13111025; doi:10.3389/fonc.2026.1807951)
Supplement: Supplementary file 1 [file Table1.docx]

| **Appendix 2: Clinical Trials Included in the Study** | | | | | | | |
| --- | --- | --- | --- | --- | --- | --- | --- |
| ID | Study Status | Study Results | PI3K inhibitor | Sex | Phases | Study Type | First Posted |
| NCT00960960 | Completed | NO | Pictilisib | All | Phase I | Interventional | 2009 |
| NCT01042925 | Completed | NO | Pilaralisib | Female | Phase I/II | Interventional | 2010 |
| NCT01082068 | Completed | NO | Pilaralisib，Voxtalisib | Female | Phase I/II | Interventional | 2010 |
| NCT01132664 | Terminated | YES | Buparlisib | All | Phase I/II | Interventional | 2010 |
| NCT01219699 | Completed | NO | Alpelisib | All | Phase I | Interventional | 2010 |
| NCT01240928 | Withdrawn | NO | Alpelisib | Female | Phase I | Interventional | 2010 |
| NCT01248494 | Completed | NO | Dactolisib，Buparlisib | All | Phase I | Interventional | 2010 |
| NCT01288092 | Withdrawn | NO | Dactolisib | All | Phase II | Interventional | 2011 |
| NCT01339442 | Completed | NO | Buparlisib | Female | Phase I | Interventional | 2011 |
| NCT01430585 | Terminated | YES | PF-04691502 | Female | Phase II | Interventional | 2011 |
| NCT01471847 | Completed | NO | Dactolisib | Female | Phase I | Interventional | 2011 |
| NCT01495247 | Terminated | NO | Dactolisib | Female | Phase I/II | Interventional | 2011 |
| NCT01513356 | Completed | NO | Buparlisib | Female | Phase 0 | Interventional | 2012 |
| NCT01572727 | Completed | YES | Buparlisib | Female | Phase II/III | Interventional | 2012 |
| NCT01589861 | Suspended | NO | Buparlisib | All | Phase I/II | Interventional | 2012 |
| NCT01610284 | Completed | YES | Buparlisib | Female | Phase III | Interventional | 2012 |
| NCT01629615 | Completed | YES | Buparlisib | All | Phase II | Interventional | 2012 |
| NCT01633060 | Terminated | YES | Buparlisib | Female | Phase III | Interventional | 2012 |
| NCT01658176 | Withdrawn | NO | PF-04691502 | Female | Phase II | Interventional | 2012 |
| NCT01740336 | Completed | NO | Pictilisib | Female | Phase II | Interventional | 2012 |
| NCT01790932 | Completed | YES | Buparlisib | All | Phase II | Interventional | 2013 |
| NCT01791478 | Active not recruiting | NO | Alpelisib | Female | Phase I | Interventional | 2013 |
| NCT01816594 | Completed | YES | Buparlisib | Female | Phase II | Interventional | 2013 |
| NCT01872260 | Active not recruiting | NO | Alpelisib | Female | Phase I/II | Interventional | 2013 |
| NCT01918306 | Terminated | YES | Pictilisib | All | Phase I/II | Interventional | 2013 |
| NCT01923168 | Completed | YES | Alpelisib | Female | Phase II | Interventional | 2013 |
| NCT01953445 | Withdrawn | NO | Buparlisib | Female | Phase II | Interventional | 2013 |
| NCT02038010 | Completed | YES | Alpelisib | Female | Phase I | Interventional | 2014 |
| NCT02058381 | Completed | NO | Alpelisib，Buparlisib | Female | Phase I | Interventional | 2014 |
| NCT02088684 | Completed | NO | Alpelisib，Buparlisib | Female | Phase I | Interventional | 2014 |
| NCT02154776 | Completed | NO | Buparlisib | Female | Phase I | Interventional | 2014 |
| NCT02260661 | Completed | NO | AZD8835 | All | Phase I | Interventional | 2014 |
| NCT02285179 | Completed | NO | Taselisib | Female | Phase I/II | Interventional | 2014 |
| NCT02340221 | Terminated | YES | Taselisib | Female | Phase III | Interventional | 2015 |
| NCT02379247 | Completed | YES | Alpelisib | All | Phase I/II | Interventional | 2015 |
| NCT02389842 | Completed | NO | Taselisib，Pictilisib | All | Phase I | Interventional | 2015 |
| NCT02390427 | Completed | NO | Taselisib | All | Phase I | Interventional | 2015 |
| NCT02404844 | Completed | NO | Buparlisib | Female | Phase II | Interventional | 2015 |
| NCT02437318 | Completed | YES | Alpelisib | All | Phase III | Interventional | 2015 |
| NCT02457910 | Terminated | YES | Taselisib | All | Phase I/II | Interventional | 2015 |
| NCT02506556 | Completed | NO | Alpelisib | All | Phase II | Interventional | 2015 |
| NCT02626507 | Unknown | NO | Gedatolisib | Female | Phase I | Interventional | 2015 |
| NCT02684032 | Completed | NO | Gedatolisib | Female | Phase I | Interventional | 2016 |
| NCT02723877 | Completed | NO | Bimiralisib | Female | Phase I/II | Interventional | 2016 |
| NCT03006172 | Active not recruiting | NO | Inavolisib | All | Phase I | Interventional | 2016 |
| NCT03128619 | Terminated | NO | Copanlisib | All | Phase I | Interventional | 2017 |
| NCT03207529 | Completed | NO | Alpelisib | All | Phase I | Interventional | 2017 |
| NCT03243331 | Completed | NO | Gedatolisib | All | Phase I | Interventional | 2017 |
| NCT03377101 | Withdrawn | NO | Copanlisib | All | Phase II | Interventional | 2017 |
| NCT03386162 | Terminated | NO | Alpelisib | All | Phase II | Interventional | 2017 |
| NCT03765983 | Terminated | NO | Paxalisib | All | Phase II | Interventional | 2018 |
| NCT03767335 | Completed | YES | Izorlisib | All | Phase I | Interventional | 2018 |
| NCT03803761 | Withdrawn | NO | Copanlisib | All | Phase I/II | Interventional | 2019 |
| NCT04108858 | Terminated | YES | Copanlisib | All | Phase I/II | Interventional | 2019 |
| NCT04142554 | Withdrawn | NO | Parsaclisib | All | Phase I | Interventional | 2019 |
| NCT04191499 | Active not recruiting | YES | Inavolisib | All | Phase I/II | Interventional | 2019 |
| NCT04208178 | Active not recruiting | NO | Alpelisib | All | Phase III | Interventional | 2019 |
| NCT04251533 | Active not recruiting | YES | Alpelisib | All | Phase III | Interventional | 2020 |
| NCT04253561 | Unknown | NO | Buparlisib | All | Phase I | Interventional | 2020 |
| NCT04345913 | Active not recruiting | YES | Copanlisib | All | Phase I/II | Interventional | 2020 |
| NCT04631835 | Unknown | NO | HS-10352 | All | Phase I | Interventional | 2020 |
| NCT04802759 | Recruiting | NO | Inavolisib | Female | Phase I/II | Interventional | 2021 |
| NCT04849364 | Terminated | YES | Inavolisib | All | Phase II | Interventional | 2021 |
| NCT04856371 | Unknown | NO | CYH33 | All | Phase I | Interventional | 2021 |
| NCT05021900 | Completed | YES | Tenalisib | Female | Phase II | Interventional | 2021 |
| NCT05090358 | Active not recruiting | NO | Alpelisib | All | Phase II | Interventional | 2021 |
| NCT05134922 | Completed | NO | Gedatolisib | Female | Phase not applicable | Expanded access | 2021 |
| NCT05143229 | Active not recruiting | NO | Alpelisib | All | Phase I | Interventional | 2021 |
| NCT05306041 | Recruiting | NO | Inavolisib | All | Phase II | Interventional | 2022 |
| NCT05307705 | Active not recruiting | NO | Inavolisib | All | Phase I | Interventional | 2022 |
| NCT05332561 | Recruiting | NO | Inavolisib | All | Phase II | Interventional | 2022 |
| NCT05501886 | Active not recruiting | NO | Gedatolisib，Alpelisib | All | Phase III | Interventional | 2022 |
| NCT05504213 | Unknown | NO | HS-10352 | All | Phase I | Interventional | 2022 |
| NCT05508906 | Recruiting | NO | Alpelisib | All | Phase I | Interventional | 2022 |
| NCT05563220 | Recruiting | NO | Alpelisib | All | Phase I/II | Interventional | 2022 |
| NCT05631795 | Completed | NO | Alpelisib | All | Phase IV | Interventional | 2022 |
| NCT05646862 | Active not recruiting | NO | Inavolisib，Alpelisib | All | Phase III | Interventional | 2022 |
| NCT05708235 | Recruiting | NO | Inavolisib | All | Phase II | Interventional | 2023 |
| NCT05810870 | Recruiting | NO | Serabelisib | All | Phase II | Interventional | 2023 |
| NCT05894239 | Recruiting | NO | Inavolisib | All | Phase III | Interventional | 2023 |
| NCT05966584 | Terminated | YES | Alpelisib | All | Phase II | Interventional | 2023 |
| NCT05967286 | Withdrawn | NO | Alpelisib | All | Phase II | Interventional | 2023 |
| NCT06189209 | Recruiting | NO | Tenalisib | Female | Phase II | Interventional | 2024 |
| NCT06239467 | Recruiting | NO | OKI-219 | All | Phase I | Interventional | 2024 |
| NCT06757634 | Recruiting | NO | Gedatolisib | All | Phase III | Interventional | 2025 |
| NCT06982521 | Recruiting | NO | RLY-2608 | All | Phase III | Interventional | 2025 |
| NCT07174336 | Recruiting | NO | LY4064809 | All | Phase III | Interventional | 2025 |
